# Blockade of IL-6 signaling prevents paclitaxel-induced neuropathy in C57Bl/6 mice

**DOI:** 10.1038/s41419-020-2239-0

**Published:** 2020-01-22

**Authors:** Petra Huehnchen, Hannah Muenzfeld, Wolfgang Boehmerle, Matthias Endres

**Affiliations:** 1 Charité – Universitätsmedizin Berlin, corporate member of Freie Universität Berlin, Humboldt-Universität zu Berlin, and Berlin Institute of Health, Klinik und Hochschulambulanz für Neurologie, 10117 Berlin, Germany; 2 Charité – Universitätsmedizin Berlin, Freie Universität Berlin, Humboldt-Universität zu Berlin, and Berlin Institute of Health, NeuroCure Cluster of Excellence, 10117 Berlin, Germany; 3 Berlin Institute of Health (BIH), Anna-Louisa-Karsch Straße 2, 10178 Berlin, Germany; 4 Charité – Universitätsmedizin Berlin, Freie Universität Berlin, Humboldt-Universität zu Berlin, and Berlin Institute of Health, Center for Stroke Research Berlin, 10117 Berlin, Germany; 5 German Center for Neurodegenerative Diseases (DZNE), Berlin, Germany; 6 German Center for Cardiovascular Research (DZHK), Partner Site Berlin, Berlin, Germany

**Keywords:** Neurodegeneration, Neuropathic pain, Neurotoxicity syndromes

## Abstract

The microtubule-stabilizing agent paclitaxel frequently leads to chemotherapy-induced peripheral neuropathy (CIN), which further increases the burden of disease and often necessitates treatment limitations. The pathophysiology of CIN appears to involve both “upstream” effects including altered intracellular calcium signaling and activation of calcium dependent proteases such as calpain as well as subsequent “downstream” neuro-inflammatory reactions with cytokine release and macrophage infiltration of dorsal root ganglia. In this study, we aimed to investigate whether these processes are linked by the pro-inflammatory cytokine interleukin-6 (IL-6). We observed that paclitaxel exposure induced IL-6 synthesis in cultured sensory neurons from postnatal Wistar rats, which could be prevented by co-treatment with a calpain inhibitor. This suggests a calcium dependent process. We demonstrate that adult C57BL/6 mice deficient in IL-6 are protected from developing functional and histological changes of paclitaxel-induced neuropathy. Furthermore, pretreatment with an IL-6-neutralizing antibody resulted in the prevention of paclitaxel-induced neuropathy in C57BL/6 mice. Electrophysiological data from our preclinical model was adequately reflected by measurements of patients undergoing paclitaxel therapy for ovarian cancer. In this cohort, measured Il-6 levels correlated with the severity of neuropathy. Our findings demonstrate that IL-6 plays a pivotal role in the pathophysiology of paclitaxel-induced neuropathy per se and that pharmacological or genetic interference with this signaling pathway prevents the development of this potentially debilitating adverse effect. These findings provide a rationale for a clinical trial with IL-6 neutralizing antibodies to prevent dose-limiting neurotoxic adverse effects of paclitaxel chemotherapy.

## Introduction

Chemotherapy-induced neuropathy (CIN) is among the most common adverse effects of chemotherapy and affects a large number of patients. In fact, affecting of up to 0.5% of the general population, CIN is more prevalent than, for example, multiple sclerosis or Parkinson’s disease^1^. Neurotoxicity not only increases the burden of disease for patients, it also limits tumor prognosis by necessitating treatment changes. Given both the population at risk, as well as the well-defined time point of chemotherapy-induced neurotoxicity, development of preventive strategies is highly desirable. However, to date a number of clinical trials with different preventive approaches have failed^2^, underlining the need for further preclinical research. Despite the frequency of this condition, the underlying pathophysiology is still only partially understood and a number of distinct, sometimes even contradictory, findings regarding intracellular processes have been published (reviewed by Carozzi et al.^3^). Increasing evidence suggests, that for many substances the pathomechanism of neurotoxicity differs from the cytotoxic mechanism of action, which holds the promise that neuroprotection can be achieved without hampering the anti-tumor effect. A number of recent studies highlight the importance of immune-mediated processes for the development of CIN in general and neuropathic pain in particular (reviewed by Lees et al.^4^). In the context of chemotherapy-induced sensory neuropathy, pro-inflammatory cytokines such as interleukin-6 (IL-6) or tumor necrosis factor-α (TNF-α) appear to be of particular relevance^5,6^. Other studies demonstrated, that knockout of gp130, a transmembrane protein involved in the signal-transduction of IL-6 and other cytokines, prevented the development of neuropathic pain in animal models of mechanical nerve injury, tumor pain, and inflammation^7,8^. Clinical studies demonstrated that increased levels of IL-6, respectively, soluble IL-6 receptor, correlated with painful CIN^9^ and patients receiving IL-6 neutralizing antibodies as part of their therapy reported less neuropathic pain compared with groups without these antibodies^10^. These findings are intriguing as IL-6 inhibition is also being tested as an (additional) antiproliferative chemotherapy^11,12^. Thus, inhibiting the IL-6 pathway may result in synergistic effects by (a) augmenting antiproliferative effects and (b) prevention of sensory neuropathy with subsequent dose-alterations.

The cytotoxic drug paclitaxel (PTX) is widely used in the treatment of solid tumors (e.g., breast and ovarian cancer, NSCLC, etc.) but at the same time often causes peripheral neurotoxicity. Its mechanism of action involves stabilization of the microtubule cytoskeleton, inhibiting the disassembly of the spindle apparatus during mitosis^13^. In clinical use, patients receiving a cumulative dose of paclitaxel above 300 mg/m^2^ body surface regularly (57–82%) develop a sensory axonal polyneuropathy^14^, which often presents with paraesthesia, allodynia, and neuropathic pain. It was previously demonstrated, that paclitaxel binds to the neuronal calcium sensor-1 (NCS-1) protein, which modulates the Inositol-1,4,5-trisphosphate receptor (InsP_3_R) inducing calcium (Ca^2+^) release from the endoplasmic reticulum in dorsal root ganglia neurons (DRGN)^15^. The subsequent activation of the Ca^2+^-dependent protease calpain was shown to contribute to cell death of DRGN in vitro^16^.

In this paper, we investigate how alterations of the intracellular Ca^2+^ homeostasis and neuro-inflammatory processes are linked by the pro-inflammatory cytokine IL-6 using both genetic as well as pharmacological interventions. These data are compared with data obtained from a clinical cohort undergoing paclitaxel therapy.

## Materials and methods

### Cell culture experiments

#### Dorsal root ganglia neurons (DRGN)

DRGN were isolated from rat neonates (P1–3) in accordance with the Charité Universitätsmedizin Berlin and state authority animal care committee’s regulations. Cells were digested in 0.28 Wünsch unit collagenase (Liberase DL, Roche, Germany) and separated by gentle trituration. Afterward, cells were passed through a 70 µm cell strainer and centrifuged with a Percoll gradient (1.019/1.038 g/ml) at 1000 g for 10 min. This typically yields a higly enriched DRGN fraction with <10% contaminating cells^17^. After centrifugation, cells were plated on poly-L-lysine/laminin-coated coverslips and maintained in Neurobasal-A media supplemented with B-27 (Gibco, Darmstadt, Germany), 0.5 mM glutamine/fresh nerve growth factor (10 ng/ml) and incubated overnight in a 95% air/5% CO_2_ humidified atmosphere in an incubator at 37 °C before treatment.

#### Cell viability assays

##### MTT

Metabolic integrity of cells was measured using the MTT assay as described by others^18^. In short, the substance is reduced by mitochondrial oxidoreductase enzymes, which leads to the formation of an insoluble formazan dye. Its concentration can be determined by measuring the absorption at 550 nm wavelength.

##### Cytotox-Fluor assay

Cells with an impaired membrane integrity show increased protease activity, which can be measured with a commercial fluorometric assay (Promega, Mannheim, Germany) following the manufacturer’s protocol. We calculated a compound measure of cell viability (live/dead ratio) from both MTT and CytoTox assays and transformed data as percent of control (vehicle treatment). Data were obtained from three biological replicates.

#### Cytokine measurements

DRGN were maintained in 96-well plates at 12,000 cells/well and treated with 100 nM PTX for 24 h and co-incubated with the calpain inhibitor MDL28170 (1 µM; Tocris Bioscience, Bristol, UK), Lithium chloride (0.5 and 1 mM; Sigma Aldrich, St Louis, MO, USA), the glycogen synthase kinase-3 (GSK-3) inhibitor A1070722 (50 nM; Tocris Bioscience, Bristol, UK) or their corresponding vehicle. After 24 h, supernatant was collected and snap frozen in liquid nitrogen. IL-6 concentrations were determined by enzyme-linked immunosorbent assay (ELISA; KRC0061, LifeTechnologies, Darmstadt, Germany) following the manufacturer’s protocol. All samples were measured as doublets (technical replicates). An appropriate standard curve ranging from 0 to 1500 pg/ml was carried according to the manufacturer’s instructions.

#### Immunocytochemistry of DRGN

Immunocytochemistry of DRGN was performed as described previously^19^. Primary antibody: anti-IL-6 (Abcam Cat# ab6672, RRID:AB_2127460, Cambridge, UK); Secondary antibody: goat anti-rabbit IgG-Alexa 488 (all: LifeTechnologies, Darmstadt, Germany). Specimens were visualized on a Leica TCS SPE confocal microscope (RRID:SCR_002140, Leica Wetzlar, Germany).

#### Western blot

DRGN were treated with 100 nM PTX or VEH for 24 h. Cell fractions were isolated with the cell fractionation kit HT (ab109718, Abcam, Cambridge, UK) according to the manufacturer’s protocol. Proteins were resolved by SDS-PAGE on a 4–20% Mini-Protean TGX Precast Gel (Bio-Rad, Munich, Germany) and transferred to a PVDF membrane (Immobilon-P, Merck Millipore, Darmstadt, Germany) and immunoreactive bands were visualized using standard methods. Antibodies used were as follows: anti-NF-κB p65 (ab16502, 1:1000; RRID: AB_443394), anti-IκBα (Abcam ab 12134, 1:1000; RRID: AB_298873, all Abcam, Cambridge, MA), and anti-GAPDH (Millipore Cat# MAB374, RRID: AB_2107445, 1:500, Merck, Darmstadt, Germany) was used as loading control. Detection of primary antibodies was performed with donkey anti-mouse (LI-COR Biosciences Cat# 926-32222, RRID: AB_621844; Li-Cor Biosciences, Lincoln, USA) and donkey anti-rabbit (LI-COR Biosciences Cat# 925-32213, RRID: AB_2715510; Li-Cor Biosciences, Lincoln, USA) secondary antibodies. Secondary antibodies were visualized with a Li-COR Odyssey CLx western blot imager (Li-Cor Biosciences, Lincoln, USA). All Western blot experiments were replicated thrice.

#### Animal experiments

##### Animal numbers, housing conditions and study approval

A total of 52 9-week-old male C57BL/6J mice (RRID: IMSR_JAX:000664) and 20 8–12-week-old male IL-6 knockout animals^20^ backcrossed on a C57BL/6J background for >10 generations were used for this study. Upon arrival in the animal facility, mice were assigned to cages with the help of randomly generated numbers. Mice were housed in groups of four to five animals and allowed food and water ad libitum. The animals were maintained on a 12:12 h light/dark cycle (7 am–7 pm). Behavioral testing was conducted between 10 am and 6 pm. If an injection was administered on the same day as behavior tests, it was administered only after all testing had been completed. The general well-being of the mice was assessed each day and weight was recorded before and 24 h after drug administration. For each drug a corresponding control group treated with the appropriate vehicle was included. This study was approved by the local official animal ethics committee prior to the execution of the experiments. All animal procedures were performed in accordance with government and the Charité Universitätsmedizin Berlin animal care committee’s regulations.

##### Drug injection protocol, sample sizes, and methods of randomization and blinding

Solutions of paclitaxel (PTX; Toronto Research Chemicals, #P132500), IL-6 neutralizing antibody (IL-6-AB; R&D Systems Cat# MAB406; RRID: AB_2233899) and control IgG (R&D Systems Cat# 6-001-A; RRID: AB_10144734) were prepared fresh before each treatment. All substances were injected intraperitoneally (i.p.); animals in the control groups received an injection of vehicle (VEH) as described below. Sample size calculations were done prior to the execution of in vivo experiments. We determined group sizes based on the effect size observed in experiments conducted previously with PTX^21^, with a desired power of 0.8 and an alpha level of 0.05 using G*Power software (Heinrich Heine Universität, Germany; RRID: SCR_013726)^22^. The calculated sample size with two different groups (PTX/VEH) per genetic background (wild type (WT)/IL-6 knockout (IL-6^−/−^)) was *n* = 10 mice per group. For the experiment with IL-6 neutralizing antibodies, we used the effect size observed for electrophysiological measurements from the first experiment in the knockout mice to calculate the group size, which was *n* = 8 mice per group.

Paclitaxel (PTX) was dissolved in Cremophor EL:Ethanol (1:1) with a concentration of 6 mg/ml and diluted in sterile 0.9% NaCl solution to a final concentration of 0.1 mg/ml. Cremophor EL:Ethanol (1:1) was diluted in sterile 0.9% NaCl solution to a final concentration of 1.6%. Mice received four intraperitoneal injections of 1 mg/kg bodyweight (BW) PTX or VEH on alternating days (cumulative dose 4 mg/kg BW PTX). Lyophilized antibodies were reconstituted with sterile PBS at 0.5 mg/ml and injected once prior to the first PTX injection at a dose of 5 mg antibody per kg BW. The commercial rat anti-mouse-IL-6 antibody MAB406 by R&D Systems, has been extensively validated in preclinical mouse studies including the nervous system. Published reports use (among others) doses from 1 mg/kg bodyweight (BW)^23^ to 5 mg/kg BW^24^ as well as doses in-between^25,26^. As we opted to replicate the finding of IL-6 knockout mice, we used the higher dose of 5 mg/kg BW.

The investigators conducting the behavior experiments as well as histological analysis were blinded throughout the entire process including statistical analysis.

#### Behavior analysis

Animals were familiarized with the investigator by handling of animals for 5 consecutive days prior to start of the experiment. During the experiments, a blinded experimenter randomly selected cages and animals. A dedicated laboratory with soundproof chambers was used for all behavior tests.

##### RotaRod

Motor coordination was assessed using the rotarod performance test. Mice were placed on a rotating rod in individual compartments, with walls on both sides and in front of them (TSE Systems GmbH, Bad Homburg, Germany). The speed of the rotating rod gradually increased, starting at four rpm and reaching a maximum speed of 40 rpm in 300 s. Latency for the animal to fall off the rod was recorded by a floor sensor. Training was carried out for 4 days with three trials per day, with a daily increase in the maximum time spent on the rod from 70 s per trial on day 1 to 300 s per trial on day 4, to allow mice to learn the task. During training, mice that fell off the rod within the designated time were gently placed back on the rod. Mice were brought back to their home cage from the moving rod only to prevent animals from exhibiting dropping behavior. The baseline was recorded on the last day of training by measuring the initial latency to fall off the rod. Follow-up testing was done at 7 and 13 days after initial PTX treatment to determine if motor coordination was affected. For each time point the results from three trials were averaged.

##### Von Frey hair test

Mechanical allodynia was measured with an electronic von Frey hair test as described by other groups^27^. The mice were placed under an inverted plastic cage with a wire-mesh floor. Investigators were trained to apply semi-flexible filaments to the center of the hind paws, gradually increasing pressure, for ~5 s. Poking either hind paw evoked a flexion reflex followed by a clear withdrawal response. The force applied to produce a withdrawal response was determined with a hand-held force transducer fitted with a 0.5 mm^2^ polypropylene tip (IITC, Woodland Hills, CA). The mechanical withdrawal threshold in grams was automatically recorded when the paw was withdrawn. The maximum pressure applied was 10 g. Four time points were recorded (baseline, days 7 and 13) and per time point the results from three trials were averaged.

#### Electrophysiology

Nerve conduction velocity (NCV) and sensory nerve action potential amplitudes (SNAP) of the caudal nerve were recorded in isoflurane anesthesia (1.3–1.7% in 50% O_2_) with a 2-channel portable electromyography and nerve conduction system (Neurosoft 3102evo, Schreiber & Tholen Medizintechnik, Stade, Germany). The protocol was adapted from ref. ^28^. In brief: Stimulation electrodes were placed at the base of the tail with the recording electrodes ~5 cm distal. A ground electrode was placed in-between the stimulation and recording electrodes. The intensity of the stimulus was gradually increased and supramaximal stimulation intensity level determined, Afterward, 50 serial stimuli were applied with supramaximal stimulation intensity and averaged to measure SNAP amplitude and NCV at three time points (baseline, days 7 and 14).

#### Histology

At the end of the experiment, animals were anesthetized with ketamine/xylazine and decapitated. Subsequently, spinal cord (thoracic and lumbar section) sciatic nerves and lumbar dorsal root ganglia were harvested.

##### Semi-thin sections of the sciatic nerve and assessment of nerve morphometry

Sciatic nerve samples from the mid-thigh were obtained from all animals, fixed in 2.5% glutaraldehyde and stained with osmium tetroxide prior to embedding in solvent-free, modified bisphenol A epoxy resin. Light microscopy was performed on 0.5 µm sections which had been stained with toluidine blue. Images of semi-thin sections were captured on a Leica DMRA microscope (Leica, Wetzlar, Germany) equipped with a PL-APO ×100 oil immersion objective using a Retiga 2000 CCD camera (Qimaging, Surrey, BC, Canada). Combinations of subimages was done automatically with MCID Core software (InterFocus Imaging Ltd, Cambridge, UK) to an image of the entire sciatic nerve. The area was measured with Fiji software^29^. Morphometrical analysis was performed by automated segmentation with a Matlab routine^30,31^, which allowed correcting misidentified axons. Fiber density per mm^2^ was calculated. In the experiments with IL-6^−/−^ animals, two samples from the vehicle and one from the PTX group showed strong artifacts from dissection and had to be excluded for technical reasons (segmentation not possible).

#### IL-6 levels in spinal cord

Thoracic and lumbar spinal cords from treated mice were snap frozen in liquid nitrogen and pulverized using hammer and pistil. Pulverized spinal cord was weighed and 1 ml/100 mg MSD Tris Lysis Buffer (R60TX-3) was added according to the manufacturers’ protocol. Samples were passed six times through a 25-gauge needle and kept on ice for 25 min prior to centrifugation at 14,000 rpm for 10 min at 4 °C. Supernatant was immediately snap frozen in liquid nitrogen and analyzed with an commercial ELISA system (U-PLEX Mouse IL-6, manufacturer #B22TX-2) according to the manfacturers instuctions on a Sector S 600 reader (All: Meso Scale, Rockville, Maryland).

### Patient data

#### CICARO study

the Charité Universitätsmedizin Berlin ethics committee (EA04/069/14) approved the CICARO study. All patients gave written informed consent to participate in the study. The study was registered at clinicaltrials.gov, identifier: NCT02753036. Eligible patients suffering from ovarian cancer were enrolled before the start of combination chemotherapy with paclitaxel (6 × 175 mg/m^2^ body surface area) and carboplatin (AUC 5) +/− bevacizumab after written informed consent. Patients underwent clinical and electrophysiological testing before and after chemotherapy using the Neurosoft 3102evo portable nerve conduction system (Schreiber & Tholen Medizintechnik, Stade, Germany). These data were used to calculate the reduced “Total Neuropathy Score”, which integrates clinical and electrophysiological findings according to previously published protocols^32^. At the same time points serum was obtained for analysis.

#### High-sensitivity IL-6 ELISA

IL-6 was measured in serum samples of patients using a high-sensitivity IL-6 ELISA kit according to the manufacturer’s instructions (Thermo Fisher Scientific Cat# BMS213HS, RRID: AB_2575453, Thermo Fisher, Waltham, USA). Lower limit of detection (LLOD) according to the manufacturer 0.08 pg/ml.

### Data processing and statistical analysis

Data processing and analysis was completed before unblinding of researchers. Gaussian distribution of datasets was checked prior to statistical analysis using Shapiro-Wilk normality test. Statistical analysis was performed using Prism v6.0 (GraphPad Software, San Diego, CA; RRID: SCR_002798). Normally distributed data were analyzed using unpaired two-sided *t*-tests (two groups), ordinary one-way ANOVA with Tukey post hoc analysis for multiple comparisons (≥3 groups), or ordinary two-way ANOVA and Tukey post hoc for multiple comparisons (two variables) and is shown as mean ± sem. Not normally distributed data were analyzed with Mann–Whitney-U Test (2 groups) or Kruskal–Wallis test with Dunn’s post hoc analysis for multiple comparisons (≥3 groups) and is depicted as median with interquartile ranges. Variance was similar between groups in a given statistical analysis. *p* < 0.05 was considered statistically significant and is depicted by an asterisk (NS: not significant). ARRIVE guidelines^33^ were followed writing the paper.

## Results

**Fig. 1:**
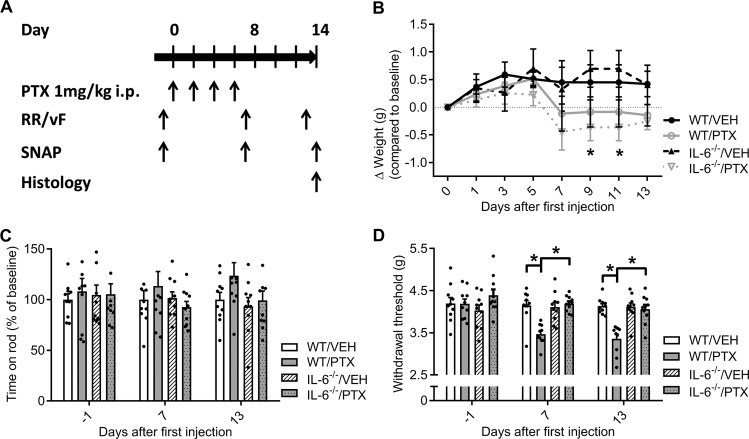
Effects of paclitaxel (PTX) treatment in wild-type and IL-6 knockout mice on motor function and mechanical allodynia. **a** Schematic overview of paclitaxel-induced neuropathy: Adult male C57Bl/6J wild-type (WT) and IL-6 knockout (IL-6^−/−^) mice were injected intraperitoneally with 1 mg per kg bodyweight (BW) paclitaxel (PTX) or the corresponding vehicle (VEH) cremophor EL:Ethanol (1:1) four times on alternating days (cumulative dose of 4 mg per kg BW PTX). Locomotor function and mechanical withdrawal threshold were assessed with the RotaRod (RR) and von-Frey (vF) test on days 7 and 13; sensory nerve action potential (SNAP) of the caudal nerve was measured on days 7 and 14 after the initial PTX application. Tissue samples for histology were obtained in terminal anesthesia on day 14. **b** PTX treated mice developed a slight weight loss over the course of the treatment, which was only significant in the PTX/IL-6^–/–^ group compared with the VEH treated IL-6^–/–^ mice. **c** Locomotor function remained unaltered after PTX application in WT and IL-6^–/–^ mice. **d** PTX treated WT mice developed a significant reduction of the mechanical withdrawal threshold indicative of mechanical allodynia, while IL-6^–/–^ mice were protected from these changes. Error bars depict SEM. Statistical analysis: **b**–**d** Two-way ANOVA with Tukey post hoc analysis of *n* = 10 mice/group; *p < 0.05, NS not significant.

We used a previously established animal model of PTX-induced neuropathy to study the role of IL-6 in the pathogenesis of PTX-induced neuropathy^21^. Adult 8–12-week-old male IL-6 knockout mice (IL-6^−/−^) and adult 9-week-old male C57Bl/6 mice were randomly assigned to two different treatment groups and received either four intraperitoneal (i.p.) injections of 1 mg/kg bodyweight (BW) PTX or vehicle (VEH) on alternating days (cumulative dose of 4 mg/kg BW PTX). Mice were assessed for behavioral and electrophysiological signs of neuropathy on days 7 and 13 after initiation of chemotherapy treatment (Fig. 1a).

### Role of IL-6 in the development of paclitaxel-induced neuropathy

**Fig. 2:**
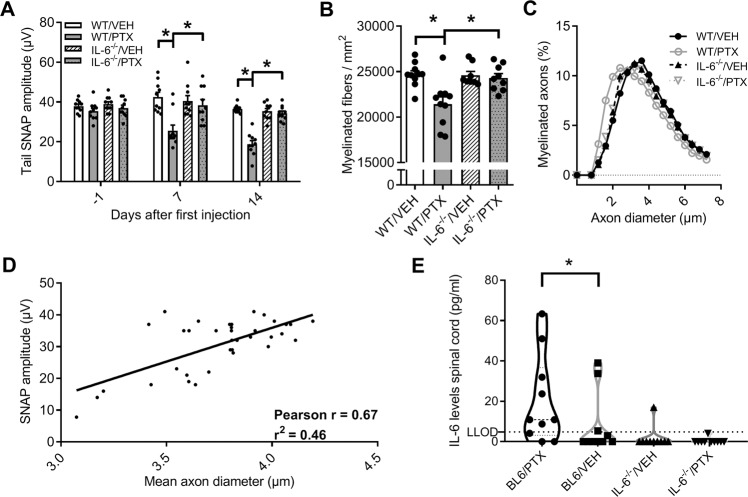
Electrophysiology and histology of paclitaxel-induced neuropathy in IL-6 knockout vs. wild-type mice. **a** PTX treated WT mice developed a significant reduction of the sensory nerve action potential (SNAP) amplitude, whereas IL-6^–/–^ mice receiving PTX showed no alterations in electrophysiological measurements. **b** Semiautomatic quantification of semi-thin sections of the sciatic nerve revealed a decrease in fiber density after PTX treatment in WT mice, while no changes could be detected in IL-6^–/–^ mice after PTX therapy. **c** PTX treatment lead to a loss of larger myelinated fibers in WT mice, while IL-6^–/–^ mice showed no shift in fiber distribution. **d** We observed a moderate positive correlation between mean axon diameter and SNAP amplitude. **e** Measurement of IL-6 concentrations in spinal cord lysates revealed significantly higher IL-6 concentrations in paclitaxel-treated wild-type mice (LLOD: Lower limit of detection). Error bars depict SEM. Statistical analysis: **a**, **b** two-way ANOVA with Tukey post hoc analysis of *n* = 10 mice/group, **d** Pearson correlation of *n* = 37 mice; **e** one-way ANOVA with Holm-Sidak post hoc analysis. *p < 0.05, NS not significant.

Many patients suffering from CIN report allodynia, which in animals can be assessed by measuring the mechanical withdrawal threshold with the von Frey method. In a first step, we were interested to see whether IL-6 deficient mice develop mechanical allodynia following PTX therapy. The treatment was generally well tolerated in both wild-type (WT) and IL-6^–/–^ mice. PTX injected mice showed a slight weight reduction, which was significant in IL-6^–/–^ mice on days 9 and 11 with an average weight loss of 0.5 ± 0.3 g compared with baseline (two-way RM ANOVA, F_(7, 252)_ = 3.701, *p* = 0.0151; Fig. 1b). Both WT and IL-6^–/–^ mice treated with PTX showed no signs of motor dysfunction (two-way ANOVA, F_(6, 72)_ = 0.7054, *p* > 0.1560; Fig. 1c). In line with previous studies, WT mice injected with PTX developed a significant reduction of the mechanical withdrawal threshold to tactile stimuli as indication of mechanical hypersensitivity (two-way ANOVA, F_(6, 72)_ = 4.404, *p* < 0.0001; Fig. 1d). IL-6^–/–^ mice treated with PTX showed no alterations of the mechanical withdrawal threshold compared with control animals and baseline. When we measured the sensory nerve action potential (SNAP) after PTX treatment, we observed no changes of SNAP amplitude or nerve conduction velocity (NCV) in IL-6^–/–^ mice. In comparison, PTX treated WT animals developed a distinct reduction of SNAP amplitude, but not NCV, indicative of a sensory axonal neuropathy commonly found in CIN patients (two-way ANOVA, F_(6, 72)_ = 4.151, *p* < 0.0001; Fig. 2a). Histological analysis of the sciatic nerve confirmed the results from the electrophysiological measurements showing a decrease of fiber density in the WT/PTX group, but not IL-6^–/–^ mice treated with PTX (two-way ANOVA, F_(1, 33)_ = 6.690, *p* = 0.0051; Fig. 2b). We observed no changes in axon diameter between animals of the IL-6^–/–^/PTX and control groups while PTX treatment resulted in a loss of larger myelinated fibers in WT mice (Fig. 2c), correlating positively to the reduction of SNAP amplitude (Pearson correlation, *r*^2^ = 0.46; Fig. 2d). To assess IL-6 concentrations in neuronal tissues, we used an ELISA to measure IL-6 concentrations in pulverized thoracic and lumbar spinal cord, which contains projections from dorsal root ganglion neurons. We observed significantly higher IL-6 levels in PTX treated wild-type animals (one-way ANOVA, F_(3, 36)_ = 4.55; *p* = 0.0084; Fig. 2e) compared with vehicle treated mice. With the exception of one (technical) outlier, no IL-6 was detected in IL-6 knockout animals. In summary, these results demonstrate that IL-6 knockout mice are protected from PTX-induced neuropathy.

### Dorsal root ganglia neurons release IL-6 upon paclitaxel exposure

**Fig. 3:**
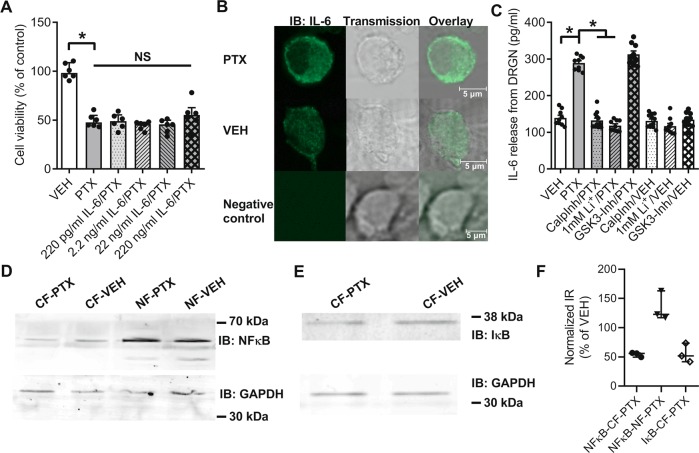
Paclitaxel induces IL-6 production in dorsal root ganglia neurons (DRGN). **a** Exposure of DRGN to 100 nM PTX for 24 h leads to a marked decrease of cell viability. Increasing concentrations of IL-6 had no additive toxic effect on DRGN cell viability. **b** Cultured DRGN from postnatal rats treated with 100 nM PTX or Vehicle (VEH) for 24 h show IL-6 immunoreactivity (first and second row). Omission of the primary antibody abolished the signal (“negative control”, third row). **c** Elevated IL-6 concentrations were detected in the supernatant of PTX treated DRGN after 24 h exposure (100 nM). Co-incubation with the calpain inhibitor MDL28170 (CalpInh; 1 µM) as well as lithium chloride (Li^+^; 1 mM), but not the glycogen synthase kinase-3 inhibitor A1070722 (GSK-3-Inh; 50 nM), maintained IL-6 levels at control values after PTX exposure. **d** DRGN were treated for 24 h with 100 nM PTX or vehicle and subsequently underwent fractionated extraction of cytosolic (CF) and nuclear (NF) proteins. Western blots of these fractions revealed a decrease of nuclear factor-κB (NF-κB) in the cytosolic and an increase in the nuclear fraction after PTX treatment compared with VEH. **e** Western blot of the inhibitor of NF-κB (IκB) shows reduced immunoreactivity after PTX treatment in the cytosolic fraction. **f** For comparison immunoreactivity normalized to the loading control was set into relation with vehicle treatment (VEH = 100%) from three experiments. This analysis supports the observation that PTX treatment leads to a decrease of cytosolic IκB and a translocation of NF-κB from the cytosol to the nucleus. Error bars depict SEM. Statistical analysis: **a**, **c** Data obtained from *n* = 3 biological replicates analyzed with (**a**) Kruskal–Wallis with Dunn’s post hoc test and (**c**) one-way ANOVA with Tukey post hoc analysis; *p < 0.05, NS not significant.

Given the pivotal role of IL-6 in the development of PTX-induced neuropathy, we were interested which effects IL-6 mediates in vitro. In order to determine whether IL-6 per se modifies PTX-induced toxicity, we incubated enriched DRGN with increasing concentrations of IL-6 in vitro. Cell viability of DRGN was markedly reduced to 49 ± 3% after 24 h exposure with 100 nM PTX (Kruskal–Wallis, *p* = 0.0065; Fig. 3a), but co-incubation of PTX with increasing IL-6 concentrations had no additional toxic effect on DRGN. We thus hypothesized that PTX exposure may induce IL-6 production in DRGN, which in turn could attract immune cells triggering an intercellular effect that amplifies PTX toxicity to DRGN^34^. Indeed, when we used immunocytochemistry to stain IL-6 in cultured DRGN treated for 24 h with 100 nM PTX or vehicle of PTX, we observed IL-6 immunoreactivity in both conditions (Fig. 3b) and staining in vehicle treated cells appeared less intense. As demonstration of immunoreactivity does not necessarily correlate with biologically relevant IL-6 secretion and quantification of immunocytochemistry is imprecise, we used an ELISA assay to assess IL-6 release from cultured DRGN and observed a strong increase of IL-6 secretion in PTX treated cultures (Fig. 3c). We previously demonstrated that PTX binds to the NCS-1 protein, which in its Ca^2+^ bound conformation positively modulates the InsP_3_R^15^. This leads to an efflux of Ca^2+^ from the endoplasmic reticulum in DRGN resulting in the activation of the Ca^2+^ dependent protease calpain^16^. In addition, it was shown that the PTX-induced interaction of NCS-1 with the InsP_3_R can be specifically inhibited by lithium ions (Li^+^) in vitro^35^, resulting in the successful prevention of PTX-induced neuropathy in vivo^36^. We hypothesized that inhibition of calpain and/or blocking the NCS-1/InsP_3_R interaction with Li^+^ might affect IL-6 production of DRGN. Indeed, IL-6 levels remained at control levels after co-incubation of PTX with the calpain inhibitor MDL28170 (1 µM) as well as Li^+^ (1 mM) (one-way ANOVA, F_(11, 90)_ = 41.61, *p* < 0.0001; Fig. 3c). Inhibition of glycogen synthase kinase-3 (GSK-3), on the other hand, a known target of Li^+^, did not prevent IL-6 release from DRGN (Fig. 3c). Our results demonstrate that interference with the NCS-1/InsP_3_R/calpain cascade influences IL-6 production of DRGN. We further hypothesized that this is due to calpain-mediated degradation of the inhibitor of nuclear factor-κB (ΙκΒ)^37,38^, which permits translocation of nuclear factor-κB (NF-κB) to the nucleus. NF-κB translocation to the nucleus is known to induce transcription of inflammatory cytokines, including the IL-6 gene^39^. To test this hypothesis, cultured DRGN were treated for 24 h with 100 nM PTX or vehicle, followed by a fractionated extraction of cytosolic (CF) and nuclear (NF) proteins. Western blots of these fractions revealed a decrease of NF-κB in the cytosolic and an increase in the nuclear fraction after PTX treatment compared with VEH (Fig. 3d, f). Correspondingly, IκB, the inhibitor of NF-κB, showed reduced immunoreactivity after PTX treatment in the cytosolic fraction (Fig. 3e, f). In conclusion, our data provide evidence that PTX mediated activation of calpain via the NCS-1/InsP_3_R pathway induces production of IL-6 in DRGN by the NF-κB signaling pathway.

### Effects of pretreatment with IL-6-neutralizing antibodies on paclitaxel-induced neuropathy

**Fig. 4:**
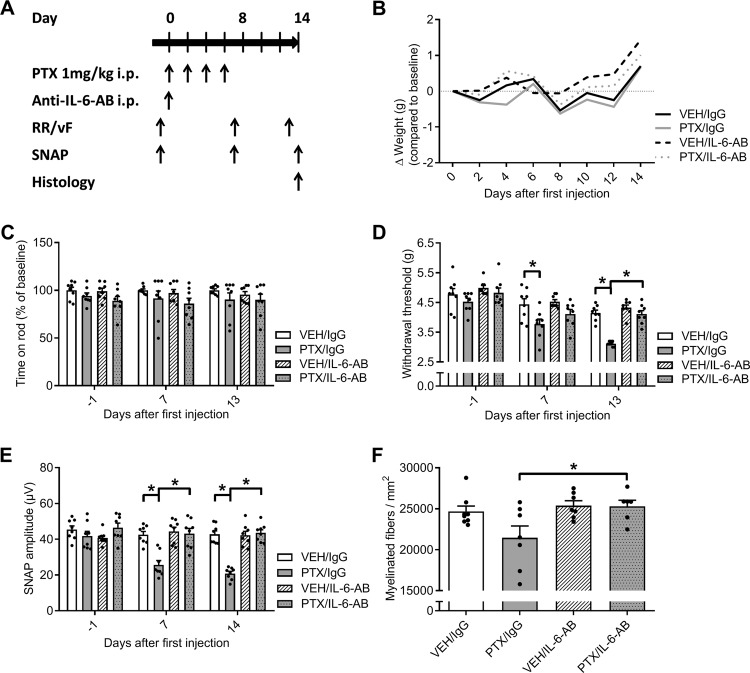
Effect of IL-6-neutralizing antibodies (IL-6-AB) on the development of paclitaxel-induced sensory neuropathy. **a** Schematic overview of preventive treatment with IL-6-AB MAB406: Adult male C57Bl/6 mice received one intraperitoneal application of the IL-6-AB MAB406 (5 mg/kg BW) or unspecific IgG prior to PTX (4 × 1 mg/kg BW) or VEH treatment. Mice were assessed for behavioral signs of neuropathy on days 7 and 13, electrophysiological measurements were performed at baseline and on days 7 and 14 after initial PTX injection. **b** There were no significant weight differences between VEH and PTX treatment in any of the groups. **c** Locomotor function remained unchanged regardless of treatment. **d** PTX treated mice, which had previously received the control IgG, developed mechanical hypersensitivity while mice of the PTX/IL-6-AB group were protected from these changes. **e** PTX treatment led to a significant reduction of the SNAP amplitude by ~50% in mice which received the control antibody (IgG), while preventive IL-6-AB application maintained axonal integrity during PTX therapy. **f** Mice with the preventive IL-6-AB treatment were protected from PTX-induced neuropathy as fiber density was comparable to control values. Error bars depict SEM. Statistical analysis: **b**–**f** two-way ANOVA with Tukey post hoc of *n* = 6–8 mice/group; *p < 0.05, NS not significant.

Next, we evaluated a preventive application of IL-6-neutralizing antibodies (IL-6-AB) in our model of PTX-induced neuropathy. Adult male C57Bl/6 mice received one intraperitoneal injection of 5 mg/kg BW IL-6-AB or unspecific IgG prior to PTX treatment. Mice were assessed for behavioral and electrophysiological signs of PTX-induced neuropathy before the start of therapy and on days 7 and 13/14 (Fig. 4a). The treatment was well tolerated and we did not observe any differences in weight over time (Fig. 4b) or signs of motor dysfunction after antibody or PTX treatment (Fig. 4c). Mice that had received the control antibody IgG in addition to PTX developed significant mechanical hypersensitivity (two-way ANOVA, F_(6, 56)_ = 2.841, *p* < 0.0001; Fig. 4d), while PTX treated mice that had previously received the IL-6-AB showed no signs of an increased sensitivity to tactile stimuli. When we measured the sensory nerve action potential of the caudal nerve, we observed a decrease of SNAP amplitude in PTX/IgG treated mice down to 49 ± 3% of control values (VEH/IgG) by day 14 (two-way ANOVA, F_(6, 56)_ = 8.195, *p* < 0.0001; Fig. 4e), while mice with preventive application of IL-6-AB were protected from these changes. Histological analysis confirmed that fiber density was reduced in the sciatic nerves of mice with the PTX/IgG treatment (two-way ANOVA, F_(1, 24)_ = 2.837, *p* = 0.0187; Fig. 4f). In contrast, mice of the PTX/IL-6-AB group showed no alterations in fiber density compared with VEH treated animals. In summary, these results demonstrate the protective effect of a preventive treatment with IL-6-neutralizing antibodies in an animal model of PTX-induced neuropathy.

### Measurement of IL-6 in a cohort of patients undergoing paclitaxel chemotherapy

**Fig. 5:**
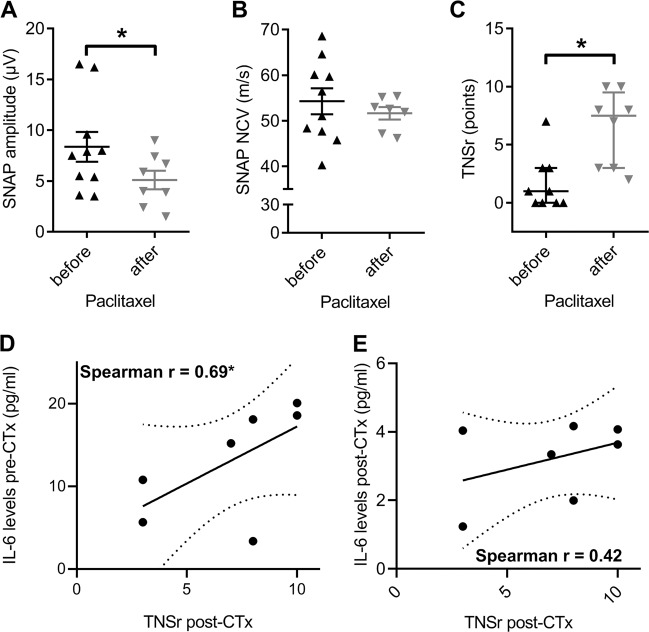
Paclitaxel-induced neuropathy in patients suffering from ovarian cancer. We analyzed data from patients suffering from ovarian cancer with regards to the development of sensory neuropathy. Patients undergoing combination chemotherapy with paclitaxel (6 × 175 mg/m^2^ body surface area) and carboplatin (AUC 5) +/– bevacizumab developed axonal sensory neuropathy, which was marked by (**a**) a significant decrease of the sural nerve sensory action potential amplitude (SNAP), while (**b**) the nerve conduction velocity was unaffected. **c** The total neuropathy score (TNSr) which integrates clinical and electrophysiological parameters showed a steep increase. Severity of paclitaxel-induced neuropathy showed a positive correlation with the serum IL-6 concentration before (**d**) more than after (**e**) chemotherapy. Error bars depict SEM. Statistical analysis: **a**, **b** *t*-test, **c** Mann–Whitney-U test, **d**, **e** Spearman correlation of seven patients; *p < 0.05, NS not significant.

In a last step, we analyzed data from the observational CICARO study with regards to the development and characteristics of sensory neuropathy. The patient, tumor and treatment characteristics of this cohort is summarized in Table 1. Patients undergoing combination chemotherapy with PTX (6 × 175 mg/m^2^ body surface area) and carboplatin (AUC 5) +/− bevacizumab developed axonal sensory neuropathy, which was marked by a significant decrease of the sural nerve sensory action potential amplitude (SNAP) by 39% compared with baseline values (*t*-test, *p* = 0.047; Fig. 5a). This decrease is comparable to our preclinical data (Fig. 2a) and no alteration of sensory nerve conduction velocity was observed (Fig. 5b) indicating that a sensory axonal polyneuropathy had developed. Chemotherapy-induced neuropathy in general and PTX-induced neuropathy in particular are known to affect the sensory nervous system (reviewed by Boehmerle et al.^40^). In the sensory nervous system, possible symptoms can be related to an abnormal gain of function with “positive symptoms”, or to a loss of function, i.e., “negative symptoms”. Electrophysiological measurements depend mainly on the integrity of large myelinated axons and thus sometimes poorly reflect the clinical situation especially concerning “positive” symptoms. Hence we chose the previously validated “Total Neuropathy Score reduced” (TNSr) as a measure for the severity of CIN as it integrates both subjective patient-reported parameters such as sensory symptoms with objective clinical findings from neurological examinations and electrophysiological measurements as primary endpoint^32^. In our patients, the TNSr, showed a steep increase by, on average, 6 points (Mann–Whitney-U test, *p* = 0.003; Fig. 5c). Previous studies reported elevated IL-6 levels in CIN patients^9^, which led us to hypothesize that severity of CIN may correlate with IL-6 serum levels. We measured IL-6 values before initiation of paclitaxel treatment and 2–4 weeks after completion of chemotherapy. At baseline before chemotherapy, the measured IL-6 serum concentrations were above the reference range in 5 out of 7 patients, while at the later time point after completion of chemotherapy, all values were within the reference range (~0.08–9 pg/ml^41^). When we plotted IL-6 serum concentrations before and after chemotherapy against the TNSr from patients treated with PTX, we observed in both cases a positive correlation: Spearman correlation, *r* = 0.69; *p* > 0.05 before (Fig. 5d) and *r* = 0.42 after chemotherapy (Fig. 5e). These findings suggest a role for IL-6 in the pathogenesis of CIN.

**Table 1:** Patient, tumor and treatment characteristics of patients analyzed.

Age, years	
Mean (SD)	57.2 (12.4)
≥65, *n* (%)	5 (50%)
Tumor stage at diagnosis, *n* (%)	
FIGO I	0
FIGO II	1 (10%)
FIGO III	9 (90%)
FIGO IV	0
Metastasis at baseline, *n* (%)	0
Baseline Karnofsky index, *n* (%)	
100	3 (30%)
90	7 (70%)
80	0
Previous neurotoxic chemotherapy, *n* (%)	0
Drop-out n (%)	2 (20%)
Number of completed chemotherapy cycles with paclitaxel, *n* (%)	
6 Cycles	6 (75%)
3 Cycles	2 (25%)
Number of completed chemotherapy cycles with carboplatin (*n*)	
6 Cycles	7 (87.5%)
3 Cycles	1 (12.5%)

## Discussion

In this study, we demonstrate an important role for IL-6 in the development of PTX-induced neuropathy. IL-6 has previously been linked to the development and maintenance of neuropathic pain^42^ and elevated IL-6 levels were detected in patients suffering from painful CIN^9^. In our study, IL-6^–/–^ mice were protected from typical hallmarks of CIN such as mechanical hypersensitivity, which we witnessed in PTX treated wild-type mice. Our data are in line with other studies showing that IL-6 is relevant for the development of mechanical allodynia^43^ and that IL-6^–/–^ mice show attenuated or delayed mechanical allodynia in models of spinal nerve lesion or chronic constriction injury^44,45^. Another study also reported beneficial effects of anti-IL-6-receptor antibodies controlling neuropathic pain after spinal cord injury in mice^46^. However, our data points to an essential role of IL-6 in the pathogenesis of CIN as IL-6^–/–^ mice treated with PTX did not develop typical behavioral, electrophysiological or histological signs of neuropathy at all. Wild-type mice on the other hand showed a reduction of SNAP amplitude that corresponds well with the observed loss of larger myelinated fibers after PTX therapy and is in line with previously published data on CIN models^47^.

**Fig. 6:**
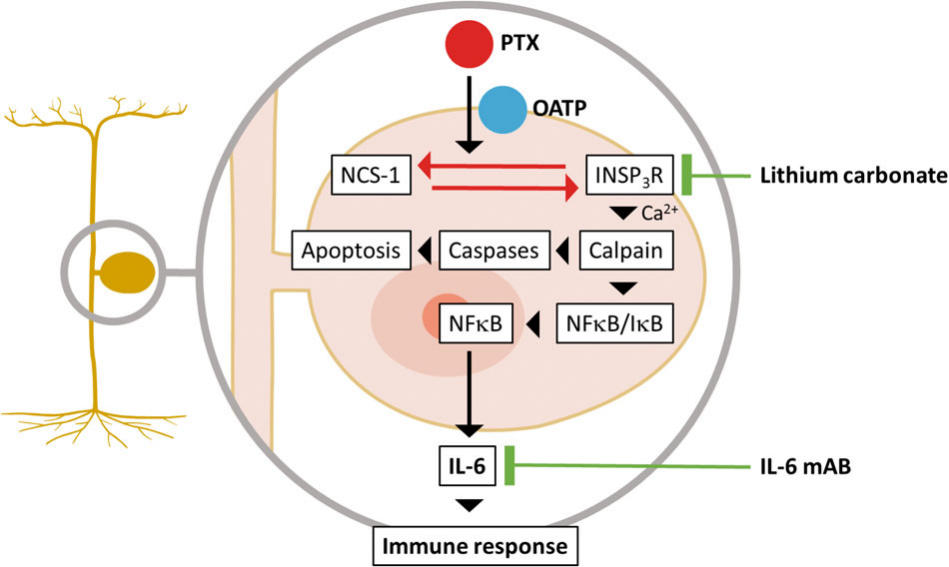
Summary graph of molecular mechanisms involved in the pathogenesis of paclitaxel-induced neuropathy and possibilities for pharmacological modulation.

In order to further elucidate the mechanistic role of IL-6 signaling in the pathophysiology of PTX-induced neuropathy, we aimed to investigate how PTX-induced Ca^2+^ dyshomeostasis^15,16^ and IL-6 synthesis are linked. One potential caveat of our study is, that even though we used and enriched culture of DRGN, there is always contamination with other cell types (Satellite glia cells, Schwann cells, Fibroblasts). We thus used immunostainings to verify the synthesis of IL-6 in DRGN. This finding was also reported previously both under physiological conditions^48^ as well as after sciatic nerve axotomy^49^, respectively, ventral root transection^50,51^. Our observation, that IL-6 production in cultured DRGN could be blocked by inhibition of calpain or the NCS-1/InsP_3_R interaction is in line with other studies, which have shown that increased levels of calpain after motor nerve injury correlated well with IL-6 upregulation in DRGN. In addition, Zang and colleagues also report that no co-staining of IL-6 with GFAP was observed, lending support to neuronal IL-6 synthesis, and that pretreatment with the calpain inhibitor MDL28170 prevented the early upregulation of IL-6 following ventral root transection^51^. In our study, MDL28170 was able to inhibit IL-6 production in DRGN after PTX exposure in vitro. In addition, we further showed that inhibition of the PTX-induced NCS-1/InsP_3_R interaction with Li^+^, upstream of calpain activation, prevents IL-6 release in vitro. Calpain activation can contribute to caspase-mediated cell death of DRGN following PTX exposure, establishing a “direct” pro-apoptotic pathway. However, it was also demonstrated, that calpain also induces the degradation of the inhibitor of NF-κB (IκB), which enables translocation of NF-κB to the nucleus (reviewed by Godwin et al.^52^). NF-κB is a known inductor of IL-6 production. When we quantified NF-κB in DRGN treated with PTX, we found increased levels of NF-κB in the nucleus and decreased levels of IκB in the cytosol, suggesting an activation of this pathway (summarized in Fig. 6).

Our observation of increased spinal IL-6 levels after paclitaxel treatment raises the question regarding the source of IL-6 measured in spinal cord tissue. Previous studies have shown neuronal IL-6 production in the spinal dorsal horn for instance after induction of mononeuropathy^53^ or upon stimulation with cytokines^54^. However, in the context of paclitaxel-induced neuropathy it was shown that spinal IL-6 synthesis is not increased as measured by rt-PCR^55^. In view of our results from cultured DRGN, the most likely source of the observed increase in spinal IL-6 concentrations after paclitaxel treatment is thus a release of IL-6 from DRGN projections.

IL-6 has many intra- and intercellular interaction partners and additional mediators of a neuro-inflammatory network are likely. For example, it was shown that chemokine X3-C ligand 1 (CX3CL1) is significantly upregulated via NF-κB-dependent H4 acetylation in PTX-induced neuropathy^56^. This is of interest as CX3CL1 seems to drive macrophage infiltration of DRGN in PTX-induced neuropathy^57^ and macrophage infiltration of DRGN was linked with the development of PTX-induced neuropathy^34^. Our data strengthen the link between the PTX-induced activation of intracellular signaling cascades in DRGN and inter-cellular mechanisms involving immune-mediated processes.

Modulating IL-6 signaling is thus a potentially valuable treatment option in the prevention of CIN. When we applied an IL-6-neutralizing antibody prior to PTX therapy, mice were protected from developing PTX-induced mechanical allodynia, electrophysiological alterations, and histological damage to nerve fibers, comparable to IL-6 knockout animals. These observations are particularly interesting as IL-6 also plays a significant role in tumor progression and tumor cell migration^58^. Early clinical data show that patients who received the IL-6-neutralizing antibody siltuximab in an experimental combination chemotherapy with the neurotoxic drug bortezomib showed less symptoms of chemotherapy-induced neuropathic pain^10^.

The magnitude and characteristics of electrophysiological changes observed in our preclinical model were comparable to measurements obtained in a cohort of ovarian cancer patients undergoing PTX chemotherapy. Although these patients also received the platinum compound carboplatin, peripheral neurotoxicity of the latter drug is secondary to PTX^59^. The correlation of serum IL-6 levels with symptoms of neuropathy, especially neuropathic pain as reported previously^9^ led us to investigate serum IL-6 levels before initiation and after completion of chemotherapy. It is important to keep in mind that plasma half-life of IL-6 is <1 h^60^, the amount of IL-6 synthesized in dorsal root ganglion neurons is likely extremely low compared with other organs and it is impossible to identify the source of IL-6 measured in serum. Intriguingly, TNSr values correlated better with IL-6 levels before than after chemotherapy, which suggests, that IL-6 levels prior to initiation of chemotherapy may be predictive for the development and severity of CIN. Clearly, larger studies are needed to confirm this hypothesis. Even though it is currently impossible to demonstrate IL-6 synthesis in sensory neurons during chemotherapy, our patient data support a link between IL-6 and the development of CIN.

In summary, the present study provides evidence indicating a much more substantial role for IL-6 in the development of CIN than was previously known. This knowledge can be potentially translated to clinical use with possible synergistic effects regarding an enhanced antiproliferative treatment and prevention of dose-limiting neurotoxic adverse effects of chemotherapy by co-treatment with IL-6 neutralizing antibodies.
